# Sex as a moderator of the relationship between childhood trauma and depression in adulthood: an analysis in a clinical sample of adult patients

**DOI:** 10.3389/fpsyt.2026.1780098

**Published:** 2026-05-20

**Authors:** Sylwia Michałowska, Magdalena Chęć, Wioletta Radziwiłłowicz

**Affiliations:** 1 Institute of Psychology, University of Szczecin, Szczecin, Poland; 2 Institute of Psychology, University of Gdańsk, Gdańsk, Poland

**Keywords:** biological sex, childhood trauma, clinical sample, depression, maltreatment, moderation analysis, peer violence, sex differences

## Abstract

**Background:**

Childhood trauma is a well-established risk factor for depression in adulthood; however, not all individuals exposed to early adversity develop depressive disorders. Biological sex has been proposed as a potential moderator of this relationship, yet findings remain inconsistent, particularly in clinical samples. The present study aimed to examine whether biological sex moderates the association between childhood traumatic experiences and depression in a clinical sample of adults.

**Methods:**

The study included 308 adults (73 patients diagnosed with depression and 235 non-clinical participants), recruited from inpatient and day psychiatric units in north-western Poland. Childhood trauma was assessed retrospectively using the Polish adaptation of the Maltreatment and Abuse Chronology of Exposure questionnaire (MACE-58 [PL]), which measures multiple forms of abuse and neglect, as well as their severity, multiplicity, and duration. Moderation analyses were conducted using binary logistic regression models implemented in the PROCESS macro (Model 1), with biological sex tested as a moderator of the relationship between childhood trauma indicators and depression.

**Results:**

Biological sex significantly moderated the association between childhood trauma indicators and the presence of a depressive disorder diagnosis across all significant models. Parental physical abuse, peer physical bullying, cumulative trauma severity, multiplicity of peer violence, and duration of maltreatment were significantly associated with depression in women, whereas no significant associations were observed in men. The strongest effects were found for repeated peer physical bullying among women.

**Conclusions:**

The findings indicate that biological sex plays a significant moderating role in the relationship between childhood trauma and depression in adulthood. Women demonstrated stronger associations between interpersonal and cumulative trauma exposure and the presence of a depressive disorder diagnosis.These results underscore the importance of sex-sensitive approaches in the assessment and treatment of depression in clinical populations.

## 1 Introduction

Early traumatic experiences in childhood, including physical, emotional, and sexual abuse, neglect, and other forms of family and peer dysfunction, constitute a significant and widespread risk factor for long-term mental health (1). A growing body of evidence indicates that experiences of child maltreatment—including physical and emotional abuse and neglect—are associated with long-term consequences for both physical and mental health, as well as with disruptions in an individual’s emotional and social development (2). Epidemiological studies indicate that exposure to trauma in early life is associated with an increased risk of developing a wide range of mental disorders in adulthood, including mood disorders (3–5), anxiety disorders (6–8), personality disorders (9–11), and substance use disorders (12, 13). The relationship between the occurrence of traumatic experiences in childhood and the later occurrence of depression is one of the best-documented associations in the psychological literature (14–17); however, the factors associated with variability in this relationship remain the subject of further investigation. Studies on experiences of child maltreatment differ in terms of the methods used to measure trauma, particularly with regard to the use of retrospective versus prospective assessments, which may explain discrepancies in the observed associations with later psychopathology (18).

In a study conducted among 349 patients with chronic depression, it was reported that as many as 75.6% of participants retrospectively reported experiences of childhood trauma, with 37% declaring multiple forms of maltreatment (19). Regression analyses indicated that although both emotional and sexual abuse were associated with a more severe course of depression, after controlling for the number of traumatic experiences, the only significant predictor that remained was the multiplicity of their occurrence (19). These results indicate that not only the occurrence of childhood trauma itself, but also its accumulation—that is, exposure to multiple forms of abuse and neglect—constitutes a significant risk factor for the development of depression. This suggests the need to consider not only the type, but also the number and severity of traumatic experiences when analyzing their long-term effects.

Early experiences of violence and neglect are particularly destructive, as they affect the developing child’s organism at both psychological and neurobiological levels. At the biological level, exposure to chronic stress or trauma early in life may lead to lasting changes in the functioning of the hypothalamic–pituitary–adrenal (HPA) axis, which has been described as long-term dysregulation of this system (20). As noted by Vinberg and colleagues (21), childhood is a period of intense brain plasticity; therefore, emotional and physical injuries may lead to persistent changes in neurotransmitter systems and in limbic structures responsible for emotion regulation. The authors emphasize that such influences may consolidate dysregulation of the stress response, increased emotional reactivity, and vulnerability to mood disorders in adulthood. As observed by Lokshina, Sheynin, and Liberzon (22), the experience of trauma is associated with enduring functional changes in brain structures. Disturbances in the functioning of these systems may contribute to the development of affective symptoms and difficulties in adaptation following traumatic experiences.

From a psychological perspective, traumatic experiences disrupt the process of forming secure attachment, the development of self-regulation mechanisms, and a sense of agency, which fosters the emergence of negative cognitive and affective patterns. As a result, childhood trauma is not merely a single event with emotional consequences, but a long-term factor shaping an individual’s biological and psychological vulnerability (21). For this reason, there has been a growing shift away from treating trauma as a categorical variable (the presence or absence of trauma in one’s life history) toward conceptualizing it as a continuum of intensity and multidimensionality of experiences (23). Most previous studies examining the association between childhood trauma and depression have relied on retrospective self-report measures such as the Childhood Trauma Questionnaire (CTQ) and have predominantly been conducted in general population samples. While these studies have consistently demonstrated significant associations between maltreatment and depressive symptoms, they often focus on selected forms of family-based abuse and neglect, with less attention paid to peer victimization, including physical bullying. Moreover, cumulative indicators of trauma exposure, such as multiplicity and duration, are less frequently analysed in clinical samples of individuals diagnosed with depressive disorders. As a result, there remains a need for research that incorporates multidimensional and chronologically sensitive measures of childhood trauma and examines these associations within clinically diagnosed populations. The present study addresses these gaps by using the MACE-58 (PL), which allows for the assessment of severity, multiplicity, and duration of diverse forms of maltreatment, including peer violence, and by testing the moderating role of biological sex in a clinical sample of adults with a depressive disorder diagnosis.

Despite the extensive body of research indicating a strong association between childhood trauma and depression in adulthood, it is still not fully clear why mood disorders do not develop in all individuals exposed to early adversity. This points to the need to identify factors that may modify this relationship. One of the key moderators may be biological sex and the psychosocial conditions associated with it. Sex is understood not only as a biological category, but also as a social construct that determines emotional expression, coping styles, and access to support (24).

In the scientific literature, a clear distinction is made between sex and gender. Sex refers to biological attributes, including chromosomal complement, hormonal profiles, and reproductive anatomy, which are typically assigned at birth. Gender, by contrast, denotes socially constructed roles, behaviours, norms, and identities associated with being a woman, a man, or a gender-diverse person, and may vary across cultural contexts. Clear differentiation between these constructs is recommended in clinical and health research to ensure conceptual precision and appropriate interpretation of findings (25). In the present study, the term sex refers to biological sex as recorded in clinical documentation. Such a perspective allows for a better understanding of how sex differences may modify the impact of trauma on the development of affective disorders. The literature indicates that biological sex and related differences in emotion regulation may influence coping styles and, consequently, the course and manifestation of depression (26). Monteiro (27) demonstrated that traumatic experiences in youth are a significant predictor of the occurrence of depression during adolescence, with particularly strong correlations observed among girls. Subsequent studies, including those conducted by Thomas and colleagues (28), have confirmed sex-specific associations between childhood trauma and negative emotional functioning, which justifies the hypothesis that sex may moderate the relationship between trauma and depression in adulthood.

Contemporary analyses (29) indicate that the pathway between childhood trauma and depression may differ between women and men. Women more often disclose depressive symptoms and seek psychological help, whereas men more frequently employ maladaptive coping strategies, which may mask symptoms of mood disorders. Vitriol and colleagues (30) draw attention to sex-related differences in the course of posttraumatic depression. In their review, the authors reported consistent findings indicating that women more often than men report experiences of emotional and sexual abuse in childhood, which may partially explain the higher prevalence of depression in this group. The authors suggest that biological factors (including differences in HPA axis reactivity and serotonergic system sensitivity), social factors (sex roles and cultural patterns of emotional responding), and psychological factors (a greater tendency toward rumination among women) may jointly contribute to sex-specific pathways of depression development following trauma. Thus, the conclusions of Vitriol et al. (30) support the concept that sex not only constitutes a risk factor differentiating the prevalence of depression, but may also moderate the relationship between childhood trauma and the severity of depressive symptoms in adulthood.

Heim et al. (31) demonstrated that men and women may respond differently to stress associated with childhood trauma, which may be related both to differences in neurohormonal regulation and to socially conditioned coping strategies. Their findings suggest that sex may not only determine the intensity of emotional responses, but also modify the relationship between trauma and the risk of developing depression in adulthood. This was also confirmed by studies conducted by Li and colleagues (32), which showed that sex moderated the relationship between childhood trauma and later emotional functioning. Trauma had a greater impact on negative patterns of thinking and behavior in women than in men. In seeking explanations for intersex differences, Hyde and colleagues (33) demonstrated that in depression these differences may be related to biological, affective, and cognitive mechanisms. Biological differences between the sexes, such as the influence of sex hormones on HPA axis functioning, neuroplasticity, and neurotransmitter signaling, may affect differential responsiveness to early stress and the development of affective disorders. Socially constructed sex roles, expectations regarding emotional expression, and access to social support may further moderate both exposure to trauma and the adaptive pathways leading to mood disorders. Consequently, sex may play a moderating role: in some individuals—and particularly in specific sex-based subgroups—the relationship between the severity of traumatic experiences and the risk of depression may be stronger or weaker.

The aim of the present study was to examine whether biological sex moderates the association between specific indicators of childhood trauma and the presence of a depressive disorder diagnosis in adulthood. The dependent variable was the presence of a depressive disorder diagnosis (clinical vs. non-clinical group). Childhood trauma was operationalised using the MACE-58 (PL) indicators, including total trauma severity (MACE SUM), multiplicity of maltreatment, duration of exposure, and selected subscales of abuse and neglect.

It was hypothesised that higher levels of childhood trauma would be associated with an increased likelihood of a depressive disorder diagnosis, and that this association would be stronger among women than among men.

## 2 Materials and methods

### 2.1 Participants

The study included a total of 308 adult participants. Of these, 73 individuals diagnosed with depression by a psychiatrist were included in the clinical group, while the remaining 235 participants constituted the non-clinical group. Participants were over 18 years of age, and their mental health status and cognitive functioning allowed for independent completion of the questionnaire. All individuals had sufficient proficiency in the Polish language, both spoken and written.

#### 2.1.1 Clinical group

Patients in the clinical group were recruited consecutively from individuals hospitalised in a Day Ward and inpatient psychiatric units in the north-western region of Poland. All consecutive patients meeting the inclusion criteria during the data collection period were invited to participate.

Inclusion criteria were:

1. age 18 years or older,
2. a current diagnosis of depressive disorder established by a psychiatrist in accordance with ICD-10 diagnostic criteria.

Exclusion criteria included acute psychotic symptoms, severe cognitive impairment, or any mental state preventing independent completion of the questionnaires.

The clinical sample consisted of 73 individuals (45 women and 28 men). Participants were predominantly individuals with secondary education (43.9%) or higher education (27.4%). The largest proportion of the sample comprised individuals who were not in a relationship (54.8%). The mean age of the clinical group was 41 years (SD = 14.67).

#### 2.1.2 Non-clinical group

The non-clinical group was recruited using a convenience sampling method from the general population. Participants in this group reported no current psychiatric diagnosis and no history of hospitalisation for mental disorders. All participants were over 18 years of age, had sufficient proficiency in the Polish language (spoken and written), and were capable of independently completing the questionnaires.

**Table 1**. Clinical sample characteristics.

**Table 1:** Sample characteristics.

Variable	Clinical sample (n = 73)	Non-clinical sample (n = 235)
Biological sex, n (%)		
Women	45 (61.6)	142 (60.4)
Men	28 (38.4)	93 (39.6)
Gender identity, n (%)		
Women	44 (60.3)	141 (60.0)
Men	28 (38.4)	93 (39.6)
Other/non-binary	1 (1.4)	1 (0.4)
Marital status, n (%)		
Married	24 (32.9)	112 (47.7)
Widowed	4 (5.5)	5 (2.1)
Single	30 (41.1)	82 (34.9)
Divorced	6 (8.2)	22 (9.4)
Other	9 (12.3)	11 (4.7)
Education level, n (%)		
Primary	8 (11.0)	9 (3.8)
Vocational	13 (17.8)	19 (8.1)
Secondary technical	4 (5.5)	32 (13.6)
Secondary	28 (38.4)	76 (32.3)
Higher	20 (27.4)	98 (41.7)
Place of residence, n (%)		
Rural area	18 (24.7)	48 (20.4)
Town/city ≤ 50,000 inhabitants	13 (17.8)	40 (17.0)
City 50,000–100,000 inhabitants	12 (16.4)	26 (11.1)
City 100,000–500,000 inhabitants	24 (32.9)	94 (40.0)
City > 500,000 inhabitants	6 (8.2)	25 (10.6)
Socioeconomic status in childhood, n (%)		
Much lower than sufficient	8 (11.1)	13 (5.5)
Lower than sufficient	15 (20.8)	50 (21.3)
Sufficient	37 (51.4)	120 (51.1)
Higher than sufficient	10 (13.9)	45 (19.1)
Much higher than sufficient	2 (2.8)	5 (2.1)
Current socioeconomic status, n (%)		
Much lower than sufficient	6 (8.5)	10 (4.3)
Lower than sufficient	7 (9.9)	18 (7.7)
Sufficient	44 (62.0)	119 (50.6)
Higher than sufficient	10 (14.1)	74 (31.5)
Much higher than sufficient	4 (5.6)	12 (5.1)
Family structure in childhood, n (%)		
Intact family	51 (69.9)	166 (70.6)
Single-parent family	19 (26.0)	41 (17.4)
Reconstituted family	3 (4.1)	27 (11.5)
Family history of mental illness, n (%)		
Yes	24 (33.3)	36 (15.3)
No	48 (66.7)	198 (84.3)

### 2.2 Procedure

Data were collected using a paper-and-pencil method. Each questionnaire packet was labeled with information about the purpose of the study and included an informed consent form for participation, information about data anonymity, and the possibility of withdrawing at any time without providing a reason. Completed questionnaires were placed in paper envelopes, sealed, and opened by the researchers only during the coding of the collected data.

To assess childhood traumatic experiences, the MACE-X questionnaire (Maltreatment and Abuse Chronology of Exposure; 34) was used in its Polish adaptation (MACE-58 [PL]; 35), which allows for the assessment of the type, duration, and timing of 10 forms of childhood abuse and neglect. Participants completed the questionnaire in the presence of a researcher, and the completed surveys were stored in a manner ensuring data confidentiality. Participation in the study was voluntary, and informed consent was obtained from all participants prior to data collection. A retrospective assessment of childhood traumatic experiences was applied in order to capture participants’ subjective experiences, which are relevant in predicting the occurrence of depression in adulthood. A meta-analysis by Baldwin et al. (18) indicates that retrospective measures of child maltreatment are more strongly associated with later psychopathology than prospective measures, which justifies the use of the MACE-58 (PL) questionnaire in the analyses.

Data collection was conducted between April 4, 2023 and November 30, 2023. In the clinical group, questionnaires were administered during hospitalisation, typically when patients’ mental state allowed for informed and voluntary participation.

Data were collected using a paper-and-pencil method. Each questionnaire packet included information about the purpose of the study, an informed consent form, and information about data anonymity and the possibility of withdrawing at any time without providing a reason. Completed questionnaires were placed in sealed envelopes and were opened by the researchers only during data coding.

Participation was voluntary, and written informed consent was obtained from all participants prior to data collection.

Given the sensitive nature of questions concerning childhood trauma, appropriate safety procedures were implemented. Participants were informed that they could discontinue participation at any time without consequences. In the clinical group, access to a treating psychiatrist or clinical psychologist was available if completing the questionnaire elicited emotional distress. In cases of observable distress, participants were offered immediate support and the opportunity to discuss their reactions with a clinician. No serious adverse events were reported during the study.

The study protocol was approved by the Bioethics Committee (approval no. KB 17/2022). All procedures were conducted in accordance with the Declaration of Helsinki and its later amendments.

### 2.3 Research tools

The MACE-58 (PL) questionnaire consists of 58 items and is divided into 10 subscales covering the following forms of experiences:

- Parental verbal abuse (PVA)
- Emotional neglect (EN)
- Physical neglect (PN)
- Parental non-verbal emotional abuse (PNEVA)
- Parental physical maltreatment (PPA)
- Witnessed interpersonal violence toward parents (WITP)
- Witnessed violence toward siblings (WITS)
- Peer verbal abuse (PEERE)
- Peer physical bullying (PEERP)
- Childhood sexual abuse (SEXA) (35).

The structure of the MACE-58 (PL) allows not only for the assessment of the occurrence of specific forms of trauma, but also for indicators such as the “total score” (MACE SUM), “multiplicity” (the number of different types of maltreatment), and “duration” (the number of years during which at least one form of maltreatment occurred), enabling the analysis of trauma as a continuum and across multiple dimensions (35). In the context of the present study, the MACE-58 (PL) was used to measure the level of childhood trauma, which was subsequently included in the analyses as an independent variable, with sex as a moderator and depression diagnosis as the dependent variable.

### 2.4 Statistical analyses

Statistical analyses were conducted using IBM SPSS Statistics version 29. Moderation analyses were performed using binary logistic regression implemented via the PROCESS macro for SPSS (Model 1; Hayes).

The dependent variable was the presence of a depressive disorder diagnosis (0 = no depression, 1 = depression), established by a psychiatrist. Childhood trauma indicators derived from the MACE-58 (PL) were entered as continuous predictors, and biological sex was included as a moderator. Sex was dummy coded (0 = women, 1 = men). Continuous trauma indicators were entered into the models in their raw (unstandardised) form and were not mean-centred prior to computing interaction terms. For each model, the main effects of the trauma indicator (X) and sex (W), as well as their interaction term (X × W), were simultaneously entered as predictors of depression diagnosis (Y).

Given the multidimensional structure of the MACE-58 (PL), separate moderation models were estimated for individual subscales as well as cumulative trauma indices (total severity, multiplicity, and duration). All planned models were tested irrespective of statistical significance.

Unstandardised logit coefficients (b), standard errors (SE), Wald statistics, odds ratios (OR), and 95% confidence intervals (CI) were reported. Model fit was evaluated using the Hosmer–Lemeshow goodness-of-fit test and Nagelkerke’s pseudo-R². To account for multiple testing, the Benjamini–Hochberg false discovery rate (FDR) procedure (q = 0.05) was applied to all regression coefficients.

Multicollinearity was assessed using variance inflation factors (VIF < 3 in all models). Influential observations were examined using Cook’s distance and leverage statistics. No influential outliers requiring exclusion were identified.

## 3 Results

Multiple binary logistic regression models were estimated to examine whether childhood trauma indicators (MACE-58 [PL]) were associated with the presence of a depressive disorder diagnosis and whether these associations were moderated by biological sex. The analyzed MACE-58 (PL) subscales encompassed both parental abuse (physical and verbal abuse, emotional and physical neglect, non-verbal emotional abuse) and peer-related experiences (peer verbal abuse, peer physical bullying), as well as witnessing violence toward parents and siblings.

### 3.1 The moderating role of sex in the relationship between trauma and the occurrence of depression

In each logistic regression model, a significant interaction between the trauma indicator and sex was found, suggesting that the impact of childhood maltreatment and abuse experiences on the depression was stronger in women than in men. Conditional effects analyses confirmed that all examined trauma indicators were significantly associated with a higher likelihood of depression among women, whereas in men these effects were non−significant (**Table 2**). Almost all key effects (trauma indicators and trauma × sex interactions) remained statistically significant after controlling for multiple testing using the Benjamini–Hochberg FDR procedure (*q* = 0.05). Only the effect of the interaction between cumulative trauma (MACE SUM) and sex did not remain significant after controlling for multiple testing using the Benjamini–Hochberg FDR procedure (*p*_BH-FDR_ = .065), and should therefore be interpreted as a trend−level finding.

**Table 2:** Sex as a moderator of the association between childhood trauma and depression.

						95% CI			95% CI OR	
Model	Variables	*b*	*SE*	*Z*	*p*	*LLCI*	*ULCI*	*OR*		*p_BH-FDR_*
Model 1	PPA	0.12	0.04	2.93	0.003	0.04	0.20	1.13	[1.04, 1.22]	0.008
	Sex	0.87	0.48	1.79	0.074	-0.08	1.81	2.39	[0.92, 6.11]	0.109
	Interaction	-0.07	0.03	-2.40	0.017	-0.12	-0.01	0.93	[0.89, 0.99]	0.030
										
Conditional effects	Women	0.05	0.02	3.08	0.002	0.02	0.09	1.05	[1.02, 1.09]	0.007
	Men	-0.01	0.02	-0.57	0.570	-0.05	0.03	0.99	[0.95, 1.03]	0.620
Model 2	PEERP	0.76	0.20	3.81	<0.001	0.37	1.14	2.14	[1.45, 3.13]	0.005
	Sex	0.44	0.32	1.38	0.168	-0.19	1.07	1.55	[0.83, 2.92]	0.221
	Interaction	-0.42	0.13	-3.21	0.001	-0.68	-0.16	0.66	[0.51, 0.85]	0.005
										
Conditional effects	Women	0.33	0.08	3.88	<0.001	0.16	0.50	1.39	[1.17, 1.65]	0.005
	Men	-0.09	0.10	-0.92	0.359	-0.29	0.11	0.91	[0.75, 1.12]	0.449
Model 3	MACE SUM	0.05	0.02	2.54	0.011	0.01	0.10	1.05	[1.01, 1.11]	0.023
	Sex	0.85	0.52	1.64	0.102	-0.17	1.87	2.34	[0.84, 6.49]	0.142
	Interaction	-0.03	0.01	-2.06	0.039	-0.06	-0.01	0.97	[0.94, 0.99]	0.065
										
Conditional effects	Women	0.02	0.01	2.70	0.007	0.01	0.04	1.02	[1.01, 1.04]	0.016
	Men	<-0.01	0.01	-0.42	0.674	-0.03	0.02	0.99	[0.97, 1.02]	0.702
Model 4	Multi PEERP	4.42	1.30	3.41	0.001	1.88	6.97	83.10	[6.55, 1064.22]	0.005
	Sex	0.20	0.30	0.67	0.503	-0.39	0.79	1.22	[0.68, 2.20]	0.599
	Interaction	-2.24	0.84	-2.67	0.007	-3.89	-0.60	0.11	[0.02, 0.55]	0.016
										
Conditional effects	Women	2.18	0.57	3.81	<0.001	1.06	3.30	8.85	[2.89, 27.11]	0.005
	Men	-0.06	0.61	-0.10	0.920	-1.26	1.14	0.94	[0.28, 3.13]	0.920
Model 5	MACE sum by Duration	0.12	0.04	2.93	0.003	0.04	0.20	1.13	[1.04, 1.22]	0.008
	Sex	0.86	0.48	1.79	0.074	-0.08	1.81	2.36	[0.92, 6.11]	0.109
	Interaction	-0.07	0.03	-2.39	0.017	-0.12	-0.01	0.93	[0.89, 0.99]	0.030
										
Conditional effects	Women	0.05	0.02	3.08	0.002	0.02	0.09	1.05	[1.02, 1.09]	0.007
	Men	-0.01	0.02	-0.57	0.570	-0.05	0.03	0.99	[0.95, 1.03]	0.620

Interaction terms represent the product of the trauma variable and sex. Simple effects indicate the associations between childhood trauma variables and depression separately for women and men. LLCI and ULCI denote the lower and upper limits of the 95% confidence interval. b – unstandardized logit coefficient (log-odds); SE, standard error; Z, Wald statistic; p, p-value; p_BH-FDR_, FDR-adjusted p-value; CI, confidence interval; OR, odds ratio.

**Table 2**. Sex as a moderator of the association between childhood trauma and depression.

**Figure 1:**
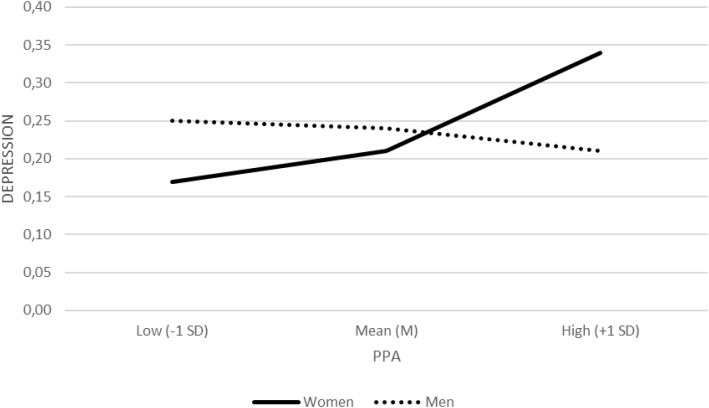
Interaction between parental physical abuse (PPA) and sex in depression (binary logistic regression). Rates of diagnosed depression are presented separately for women and men at low (−1 SD), mean (M), and high (+1 SD) levels of PPA.

Parental physical abuse (PPA) was significantly associated with depression (*b* = 0.12, *SE* = 0.04, *Z* = 2.93, *p* = .003, OR = 1.13, 95% CI [1.04, 1.22]). The interaction between PPA and sex was significant (*b* = −0.07, *SE* = 0.03, *Z* = −2.40, *p* = .017, OR = 0.93, 95% CI [0.89, 0.99]), indicating that the association between PPA and depression differed between women and men. Simple effects showed that higher levels of PPA were associated with increased odds of depression among women (*b* = 0.05, *SE* = 0.02, *Z* = 3.08, *p* = .002, OR = 1.05, 95% CI [1.02, 1.09]), whereas no significant association was observed among men (*b* = −0.01, *SE* = 0.02, *Z* = −0.57, *p* = .570, OR = 0.99, 95% CI [0.95, 1.03]) (**Figure 1**).

**Figure 2:**
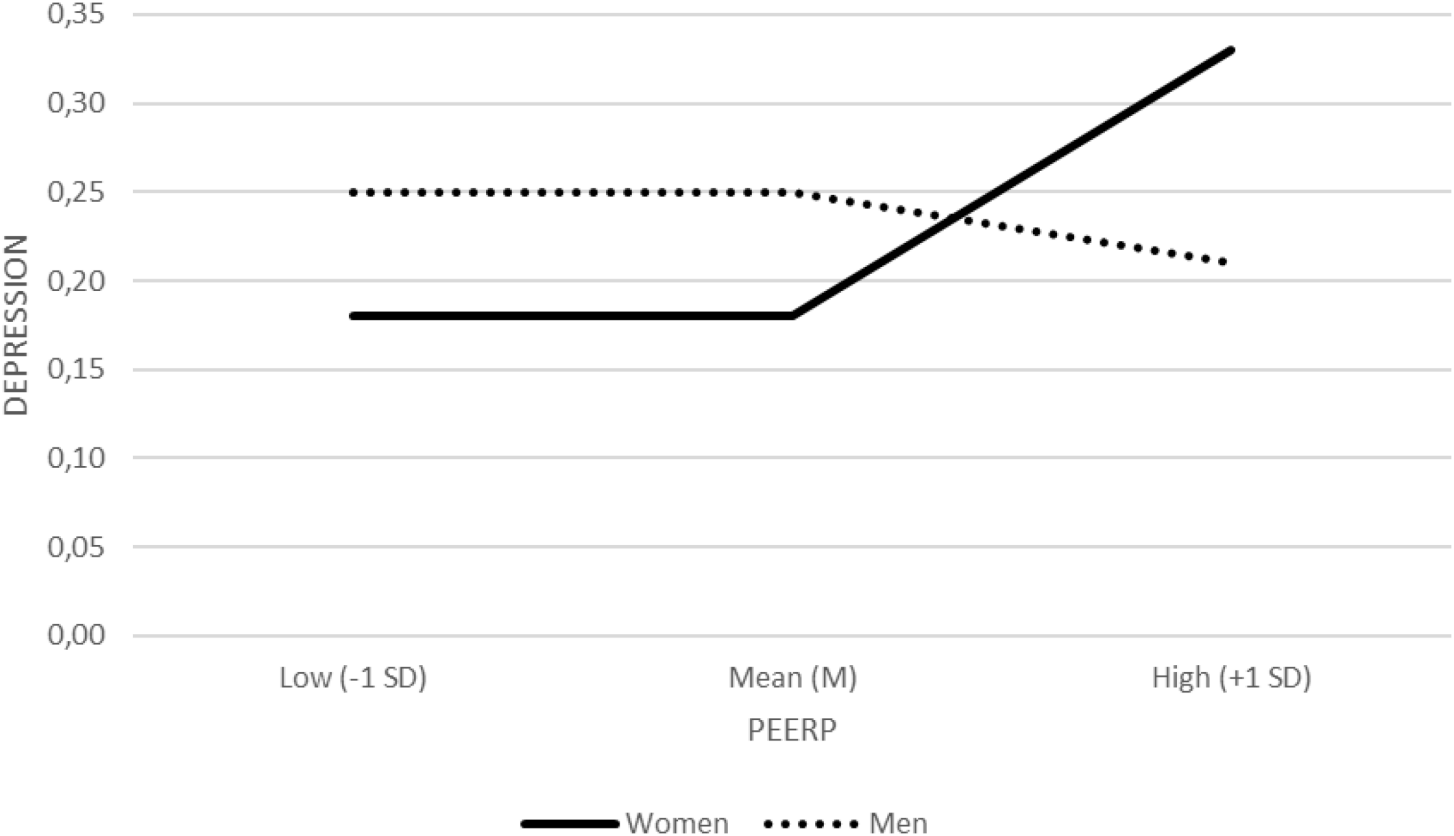
Interaction between peer physical abuse (PEERP) and sex in depression (binary logistic regression). Diagnosed depression is plotted separately for women and men at low (−1 SD), mean (M), and high (+1 SD) levels of PEERP.

Peer physical bullying (PEERP) was significantly associated with depression diagnosis (*b* = 0.76, *SE* = 0.20, *Z* = 3.81, *p* <.001, OR = 2.14, 95% CI [1.45, 3.13]). The PEERP × sex interaction was significant (*b* = −0.42, *SE* = 0.13, *Z* = −3.21, *p* = .001, OR = 0.66, 95% CI [0.51, 0.85]), suggesting a stronger trauma–depression link in women than in men. Conditional effects indicated that among women, each one−unit increase in PEERP was associated with a 39% increase in the odds of depression (*b* = 0.33, *SE* = 0.08, *Z* = 3.88, *p* <.001, OR = 1.39, 95% CI [1.17, 1.65]), whereas among men the association was non−significant (*b* = −0.09, *SE* = 0.10, *Z* = −0.92, *p* = .359, OR = 0.91, 95% CI [0.75, 1.12]) (**Figure 2**).

**Figure 3:**
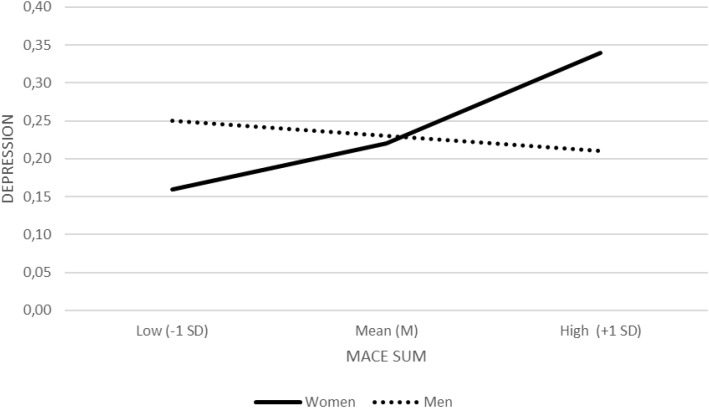
Interaction between cumulative childhood maltreatment (MACE SUM) and biological sex in predicting depression diagnosis (binary logistic regression). Predicted probabilities of depression diagnosis are plotted separately for women and men at low (−1 SD), mean (M), and high (+1 SD) levels of MACE SUM.

Model examined cumulative trauma severity (MACE SUM), which was positively related to depression (b = 0.05, SE = 0.02, Z = 2.54, p = .011, OR = 1.05, 95% CI [1.01, 1.11]). The interaction between MACE SUM and sex was significant (b = −0.03, SE = 0.01, Z = −2.06, p = .039, OR = 0.97, 95% CI [0.94, 0.99]), again indicating sex−specific patterns. Simple effects showed that cumulative trauma was associated with higher odds of depression among women (b = 0.02, SE = 0.01, Z = 2.70, p = .007, OR = 1.02, 95% CI [1.01, 1.04]), whereas no significant association was found among men (b ≈ 0.00, SE = 0.01, Z = −0.42, p = .674, OR = 1.00, 95% CI [0.97, 1.02]) (**Figure 3**).

**Figure 4:**
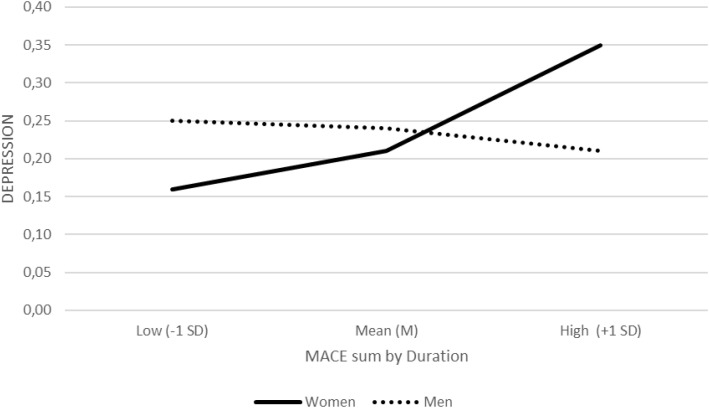
Interaction between cumulative childhood maltreatment by duration (MACE SUM by duration) and biological sex in predicting depression diagnosis (binary logistic regression). Predicted probabilities of depression diagnosis are plotted separately for women and men at low (−1 SD), mean (M), and high (+1 SD) levels of MACE SUM by duration.

Model focused on the duration of maltreatment (MACE by Duration), which was significantly related to depression (b = 0.12, SE = 0.04, Z = 2.93, p = .003, OR = 1.13, 95% CI [1.04, 1.22]). The interaction between duration and sex was significant (b = −0.07, SE = 0.03, Z = −2.39, p = .017, OR = 0.93, 95% CI [0.89, 0.99]). Simple effects showed that longer duration of maltreatment was associated with higher odds of depression among women (b = 0.05, SE = 0.02, Z = 3.08, p = .002, OR = 1.05, 95% CI [1.02, 1.09]), whereas this association was not significant among men (b = −0.01, SE = 0.02, Z = −0.57, p = .570, OR = 0.99, 95% CI [0.95, 1.03]) (**Figure 4**).

**Figure 5:**
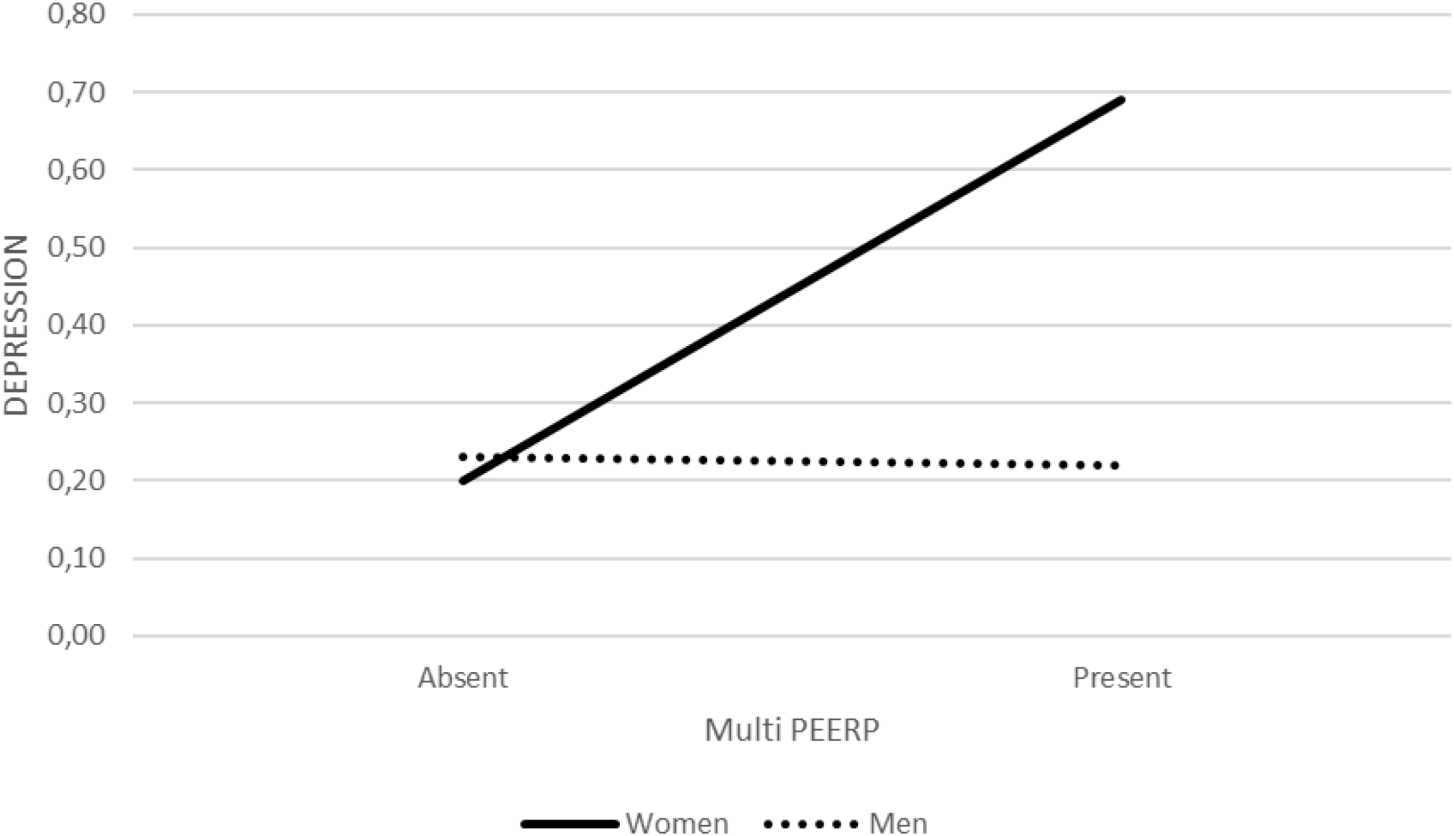
Interaction between multiplicity of peer physical bullying (Multi PEERP) and biological sex in predicting depression diagnosis (binary logistic regression). Predicted probabilities of depression diagnosis are plotted separately for women and men at low (−1 SD), mean (M), and high (+1 SD) levels of Multi PEERP.

The multiplicity of peer physical bullying (Multi PEERP) showed the strongest effect on depression (b = 4.42, SE = 1.30, Z = 3.41, p = .001, OR = 83.10, 95% CI [6.55, 1064.22]). The Multi PEERP × sex interaction was significant (b = −2.24, SE = 0.84, Z = −2.67, p = .007, OR = 0.11, 95% CI [0.02, 0.55]), indicating that the impact of multiple forms of peer violence differed markedly by sex. Among women, each additional type of peer physical bullying was associated with nearly a nine−fold increase in the odds of depression (b = 2.18, SE = 0.57, Z = 3.81, p <.001, OR = 8.85, 95% CI [2.89, 27.11]), whereas among men the association was non−significant (b = −0.06, SE = 0.61, Z = −0.10, p = .920, OR = 0.94, 95% CI [0.28, 3.13]) (**Figure 5**).

The obtained results underscore that sex plays a significant moderating role in the relationship between maltreatment experiences and a history of childhood abuse. Women exhibit a more pronounced association between parental and peer violence and depression diagnosis. This may indicate greater sensitivity of women to negative interpersonal experiences in childhood, differences in the perception and reporting of maltreatment, or a differential impact of these experiences on psychological development.

## 4 Discussion

The aim of the present study was to determine whether biological sex moderates the relationship between childhood traumatic experiences and depression in a clinical sample. The obtained results showed that sex serves as a moderator of the relationship under investigation. In all models in which a significant interaction was observed, childhood traumatic experiences were significantly associated with depression exclusively in the group of women. No statistically significant associations were revealed in men. This pattern may reflect sex-specific differences in the association between childhood trauma and depression. The absence of significant associations in men should be interpreted with caution. Several alternative explanations may account for this pattern. First, men may underreport experiences of childhood trauma, particularly interpersonal and sexual victimization, due to social norms discouraging emotional disclosure, which could attenuate observed associations. Second, childhood trauma in men may be more strongly associated with externalizing phenotypes, including substance use, aggression, or behavioral dysregulation, rather than with internalizing symptoms such as depression. Thus, the lack of significant associations in the present study may reflect differences in symptom expression rather than a true absence of trauma-related effects. Finally, the relatively small number of men in the clinical sample may have reduced statistical power to detect moderation effects in this subgroup.

Based on detailed analyses, it was revealed that physical abuse by parents in childhood was significantly associated with depression in women, whereas no such relationship was observed in men. Similar results were obtained with regard to peer violence. Physical violence by peers proved to be one of the strongest predictors of depression in the group of women, particularly when this violence was experienced repeatedly. In contrast to women, peer violence was not significantly associated with depression in men. The obtained results indicate that both parental and peer violence in childhood may have particularly negative and long-term consequences for women’s mental health.

The present study also revealed significant intersex differences in the severity, accumulation, and duration of traumatic experiences. The total score obtained on the MACE-58 questionnaire and the duration of maltreatment were significantly associated with depression only in the group of women. The strongest effects were found for indicators describing the multiplicity of peer violence, suggesting that repeated experiences of interpersonal violence in childhood may may be particularly strongly associated with depression among women. The obtained results are consistent with previous studies demonstrating the importance of multiple traumatic experiences in the development of mood disorders in adulthood (15, 19). Previous neurobiological research has shown that chronic exposure to trauma is associated with persistent changes in brain structure and functioning (36). Although the present study did not assess biological processes directly, the observed pattern of associations may be consistent with findings suggesting sex-related differences in stress responsivity. However, given the cross-sectional and retrospective design, no conclusions regarding underlying biological mechanisms can be drawn. Studies show that experiencing childhood trauma in women is associated with a different pattern of HPA axis regulation, greater emotional reactivity, and changes in brain structures responsible for the processing of emotions and stress (the amygdala and prefrontal cortex), compared to men (37, 38). These differences have been proposed as potential explanations for observed sex differences in depression prevalence, although such mechanisms were not directly examined in the present study. A meta-analysis by Zhang and colleagues (39) indicates the existence of intersex differences in the associations between childhood trauma and depression in adulthood, as well as stronger or more consistent associations in women. Similar results were obtained in a study by Assari et al. (40). It is worth noting that in men, responses to early childhood trauma more often involve symptoms of externalizing disorders or substance use–related problems (33). This may indicate the existence of sex-specific pathways in the development of psychopathology and may explain the lack of significant associations between trauma and depression in the present analyses.

In interpreting the present findings, it is also important to note the role of psychosocial mechanisms. Intersex differences in the development of depression may result from the fact that women more often than men use emotion-focused coping strategies and are more prone to rumination, which may contribute to the development or maintenance of depressive symptoms (26). In addition, interpersonal violence in childhood may promote the internalization of negative cognitive schemas related to self-worth, guilt, or helplessness in women, which play a key role in the psychopathology of depression (41). From a developmental perspective, previous research suggests that such experiences are associated with a loss of trust in others, which negatively affects developing mechanisms of emotion regulation, sense of security, and functioning in social relationships (42). Men, on the other hand, more often respond to childhood trauma through avoidance or externalization of problems (24, 29).

The present study has several limitations. The retrospective assessment of childhood trauma may have been distorted during the reconstruction of memories by the participants. It is worth noting, however, that according to the results of a meta-analysis, this type of measurement shows stronger associations with later psychopathology than prospective measurement (18). A second limitation of the study is its cross-sectional design, which precludes causal inference. Therefore, the findings should be interpreted as reflecting associations rather than causal effects. Moreover, the statistical analyses included only biological sex, without reference to gender identity or cultural context, which could allow for a more comprehensive capture of socio-cultural and individual factors moderating the relationship between trauma and depression. When discussing the limitations of the study, it should also be taken into account that the sample size, due to its clinical nature, was limited, which may have affected the precision of the estimated effects. Future studies should therefore identify mediating and moderating mechanisms of the relationship between childhood trauma and depression from a longitudinal perspective in sufficiently large clinical samples. The very large odds ratios observed for multiplicity of peer violence should be interpreted cautiously, given the relatively small sample size and wide confidence intervals. The relatively small number of men in the sample may have limited statistical power to detect interaction effects.

In summary, the results of the present study indicate that biological sex is a significant moderator of the relationship between childhood trauma and depression in adulthood in a clinical sample. As observed, women are particularly vulnerable to the long-term consequences of interpersonal and repeated trauma, which suggests the need for a systematic, sex-sensitive approach to the assessment of traumatic experiences in both the diagnosis and treatment of depression.

Recent clinical research further supports the importance of considering sex differences in depression beyond etiological factors. For example, real-world clinical data indicate that men and women may differ in suicidal and self-harming responses to esketamine treatment in treatment-resistant depression (43). Studies conducted in treatment-resistant depressive populations have demonstrated sex-related differences in clinical outcomes, including suicidality and self-harming responses to esketamine treatment (e.g., 44, 45). Emerging evidence also highlights heterogeneity in depressive phenotypes, including dissociative and depersonalization-related subtypes that may be particularly relevant in individuals with trauma histories (46). These findings suggest that sex differences may extend to symptom dimensions, treatment response, and clinical presentation. Although the present study did not examine treatment outcomes directly, the observed sex-specific associations between childhood trauma and depression diagnosis may contribute to a more refined, individualized clinical formulation that takes into account both trauma exposure and sex-related differences in depressive profiles.

Taking biological sex into account in research on depression may contribute to a more nuanced understanding of sex-related differences in the patterns of association between trauma exposure and depressive disorders.

## Data Availability

The raw data supporting the conclusions of this article will be made available by the authors, without undue reservation.
